# IRF5 regulates airway macrophage metabolic responses

**DOI:** 10.1111/cei.13573

**Published:** 2021-01-28

**Authors:** G. J. Albers, J. Iwasaki, P. McErlean, P. P. Ogger, P. Ghai, T. E. Khoyratty, I. A. Udalova, C. M. Lloyd, A. J. Byrne

**Affiliations:** ^1^ Inflammation, Repair and Development Section National Heart and Lung Institute Imperial College London London UK; ^2^ The Kennedy Institute of Rheumatology University of Oxford Oxford UK

**Keywords:** lung, macrophage, metabolism, transcription factors, virus

## Abstract

Interferon regulatory factor 5 (IRF5) is a master regulator of macrophage phenotype and a key transcription factor involved in expression of proinflammatory cytokine responses to microbial and viral infection. Here, we show that IRF5 controls cellular and metabolic responses. By integrating ChIP sequencing (ChIP‐Seq) and assay for transposase‐accessible chromatin using sequencing (ATAC)‐seq data sets, we found that IRF5 directly regulates metabolic genes such as hexokinase‐2 (*Hk2*). The interaction of IRF5 and metabolic genes had a functional consequence, as *Irf5^−/−^* airway macrophages but not bone marrow‐derived macrophages (BMDMs) were characterized by a quiescent metabolic phenotype at baseline and had reduced ability to utilize oxidative phosphorylation after Toll‐like receptor (TLR)‐3 activation, in comparison to controls, *ex vivo*. In a murine model of influenza infection, IRF5 deficiency had no effect on viral load in comparison to wild‐type controls but controlled metabolic responses to viral infection, as IRF5 deficiency led to reduced expression of *Sirt6* and *Hk2*. Together, our data indicate that IRF5 is a key component of AM metabolic responses following influenza infection and TLR‐3 activation.

## Graphical abstract

Here, we provide novel data showing that the transcription factor Interferon regulatory factor 5 (IRF5) controls cellular and metabolic responses to influenza infection and TLR3 activation in vivo. Upon pro‐inflammatory stimulation IRF5 co‐localises with open chromatin regions at metabolism‐associated genes; these interactions led to a functional consequences as IRF5 deficient airway macrophages (AMs) had an impaired ability to utilize oxidative phosphorylation. Our data highlight a novel role for IRF5 in the regulation of AM metabolic responses.

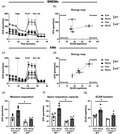

## Introduction

Airway macrophages (AMs), strategically located at the interface between the external and internal pulmonary environment, form the first line of defence against pulmonary viral infection [1]. AMs are crucial for the elimination of pulmonary viral pathogens, and defects in this activity has been shown to worsen disease outcome [2, 3]. As a critical component of pulmonary immunity, AMs are tightly controlled in order to preserve homeostasis [4, 5]. Interferon regulatory factor 5 (IRF5) is a key transcription factor involved in regulating the expression of proinflammatory cytokine responses to microbial and viral infection [6] and macrophage phenotype [6]. Previously, we have shown that IRF5 deficiency in AMs leads to enhanced remodelling and augmented T helper type 2 (Th2) responses in a murine model of allergic airways disease [7].

It has become increasingly clear that metabolic alterations underlie macrophage phenotype, and several seminal findings have shown that upon activation macrophages ﻿undergo metabolic reprogramming, which supports either pro‐ or anti‐inflammatory phenotypes [8]. Although IRF5 has been shown to be a master transcriptional regulator of macrophage phenotype [6], the role of IRF5 in controlling AM metabolic responses has not been addressed.

Integrating ChIP sequencing (ChIP‐Seq) and assay for transposase‐accessible chromatin using sequencing (ATAC)‐seq data sets we find that IRF5 directly regulates *hexokinase‐2* (*Hk2*) expression in macrophages. Importantly, the interaction between IRF5 and *Hk2* had a functional consequence, as *Irf5^−/−^* AMs had reduced maximal and spare respiratory capacity compared to wild‐type (WT) AMs. Furthermore, *Irf5* knockout animals have altered responses to influenza A virus (IAV) infection characterized by reduced inflammatory infiltrate, diminished expression of several antiviral pathways and a reduced expression of key metabolic genes, such as *Hk2*. Together, these data indicate that IRF5 is a key component of AM phenotypical, anti‐viral and metabolic processes.

## Materials and methods

### Animals

Female WT or *Irf5^−/−^* mice on a C57BL/6 background, 6‐8 weeks old, were intranasally (i.n.) infected with 50 μl of a 4·5 × 10^3^ plaque‐forming units (PFU)/ml solution of X31 (a kind gift from Andreas Wack, Crick Institute) in phosphate‐buffered saline (PBS) or PBS control, under isofluorane anaesthesia. Weight and symptoms were measured daily to monitor disease severity. Mice were deemed to have reached their humane end‐point if weight loss exceeded 25% of body weight on 2 consecutive days in accordance with our Home Office licence. Imperial College London Animal Welfare and Ethical Review Body (AWERB) approved this protocol. All surgery was performed under ketamine and sodium pentobarbital anaesthesia and all efforts were made to minimize suffering. BAL and lung cell preparations were prepared as previously described [7].

### Quantification of immunoglobulins

Paired antibodies for immunoglobulin (Ig)E, IgG1 and IgG2a (R&D Systems, Abingdon, UK) were used to measure serum and lung antibody levels, as per the manufacturer’s instructions.

### Flow cytometric analysis

Disaggregated lung (left lobe) or BAL cells were washed and preincubated with serum or Fc block (2.4G2) prior to surface staining with the following antibodies purchased from (clones in brackets): Biolegend, Inc. (San Diego, CA, USA); F4/80 (BM8), CD68 (FA‐11), inducible T cell co‐stimulator (ICOS) (C3984A), lymphocyte antigen 6 complex locus G6D (Ly6G) (1A8), CD64 (X54‐5/7.1), CD3e (145‐2C11), CD8a (3‐6.7), eBioscience, Inc. (San Diego, CA, USA); interleukin (IL)‐13 (ebio13A), IL‐17 (ebio17B7), Ly‐6G/Ly‐6C (GR‐1) (RB6‐8C5), CD11c (N418), CD45 (30‐F11), CD11b (M1/70), interferon (IFN)‐γ (XMG1.2), lineage cocktail (17A2, RA3‐6B2, M1/70, TER‐119, RB6‐8C5); eBiosciences; Ly6C (AL21), CD4 (RM4‐5), Siglec‐F (E50‐2440), T1/ST2 (RMST2‐33): abcam (Cambridge, UK): IRF5 (ab178899). Labelled cells were acquired on a BD fluorescence‐activated cell sorting LSR Fortessa (BD Biosciences, San Jose, CA, USA) and further analysed using FlowJo (Treestar, Inc., Ashland, OR, USA). Surface staining was followed by fixation and then permeabilization to allow for intracellular or intranuclear staining.

### Real‐time PCR

Total RNA was isolated using the RNeasy Plus Micro or Mini kit (Qiagen, Valencia, CA, USA) and quantified using the high‐sensitivity RNA ScreenTape kit and the 2200 TapeStation system (both Agilent, Santa Clara, CA, USA) or using the NanoDrop 1000 spectrophotometer (Thermo Fisher, Waltham, MA, USA). cDNA was generated using the high‐capacity cDNA reverse transcription kit (Thermo Fisher) and real‐time polymerase chain reaction (PCR) was performed using TaqMan gene expression probes for murine *Actb* (Mm00607939_s1), *Hprt* (Mm03024075_m1), *Hk2* (Mm00443385_m1), *Sirt6* (Mm01149042_m1) and *Irg1* (Mm01224532_m1). To determine viral load, real‐time PCR for influenza virus was performed using fast SYBR Green Master Mix (Thermo Fisher) with forward and reverse primers for influenza matrix 1 protein (*M1*; forward: TGAGTCTTCTAACCGAGGTC, reverse: GGTCTTGTCTTTAGCCATTCC) and *Actb* (forward: CTAAGGCCAACCGTGAAAAG, reverse: ACCAGAGGCATACAGGGACA). Gene expression relative *Actb* or *Actb* and *Hprt* were calculated as 2^−dCT^.

### Metabolic analysis

AMs from BAL fluid were collected by lavaging the airways of naive wild‐type or *Irf5^−/−^* mice three times with three × 1 ml of ice‐cold PBS + 5 mM ethylenediamine tetraacetic acid (EDTA) via a tracheal cannula. Bone marrow‐derived macrophages (BMDMs) were cultured in complete RPMI supplemented with 55 µM 2‐mercaptoethanol and recombinant human macrophage–colony‐stimulating factor (M‐CSF) (100 ng/ml; Peprotech, Rocky Hill, NJ, USA). Media were refreshed on day 4. On day 7, cells were harvested using ice‐cold PBS containing 5 mM EDTA. For metabolic analysis, wells of a Seahorse XFp Cell Culture Miniplate (Agilent Technologies) were treated with Cell‐Tak solution (Corning, New York, NY, USA), following the manufacturer’s instructions. AMs or BMDMs (150 000) were seeded in 100 µl RPMI complete media and treated with poly(I:C) (100 µg/ml; InvivoGen, San Diego, CA, USA) at 37°C for 24 h. Cells were washed with Seahorse assay media (Seahorse XF base medium + 1 mM pyruvate, 2 mM glutamine, 10 mM glucose) and incubated at 37°C (in the absence of CO_2_) in Seahorse assay media for 45 min–1 h. Oxygen consumption rate (OCR) and extracellular acidification rate (ECAR) were measured using the Cell Mito Stress Test kit (Agilent Technologies), as per the manufacturer’s instructions, with final well concentrations of 1 µM oligomycin, 2 µM carbonyl cyanide‐4‐(trifluoromethoxy)phenylhydrazone (FCCP) and 0·5 µM rotenone/antimycin A. OCR and ECAR measurements were made with an XFp Extracellular Flux Analyzer (Agilent Technologies) and results were analysed with Wave software version 2.6.0 (Agilent Technologies).

### ChIP‐Seq and chromatin accessibility

IRF5 ChIP‐Seq data generated from BMDMs after 2 h of lipopolysaccharide (LPS) exposure were reported previously [9]. Open chromatin regions (OCRs) across immune cell lineages/organ types were determined by assay for transposase‐accessible chromatin using sequencing (ATAC)‐seq as part of the Immunologic Genome Project (ImmGen) [10]. Overlapping IRF5/OCRs were identified using bedtools *(‐intersect)*. Chromatin accessibility scores of overlapping IRF5/OCRs were used in principal component analysis using past version 3.20 [11] and associated genes [transcription start site (TSS) and within 100 kb] were classified via great [12] and PantherDb version 14.1 (http://pantherdb.org). As we saw a clear separation of cell types along PC1, we identified the most discriminatory OCRs of the myeloid, monocyte and DC lineages by ranking OCRs by PC1 loadings and selecting the top 100. Heat‐maps of discriminatory OCRs accessibility were generated with Morpheus (https://software.broadinstitute.org/morpheus/).

### Statistical analysis

Data were analysed using Prism version 8 for Windows from GraphPad Software Inc., using Kruskal–Wallis or Mann–Whitney *U*‐tests.

## Results

### IRF5 binding is enriched at metabolic genes in macrophages

**Fig. 1:**
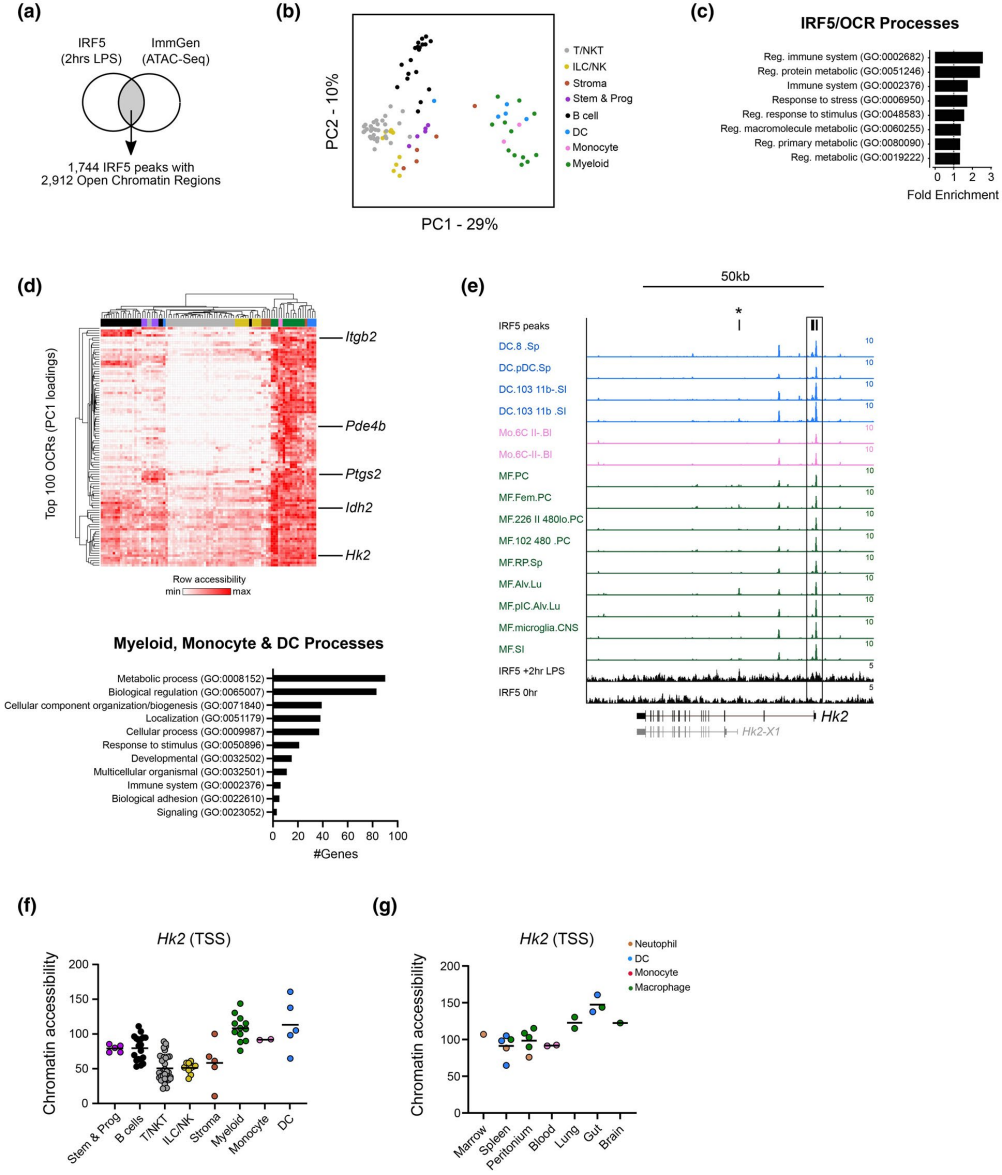
Interferon regulatory factor 5 (IRF5) binding is enriched at metabolism genes in macrophages. (a) Overlap between IRF5 ChIP sequencing (ChIP‐Seq) data from bone marrow‐derived macrophages and open chromatin regions (OCRs) across immune cell types. (b) Principal component analysis (PCA) depicting the separation of monocyte, myeloid and dendritic cells (DCs) from all other cell lineages based on chromatin accessibility of OCRs overlapping IRF5 peaks. (c) Pathway analysis of IRF5/OCRs‐associated genes indicating a predominance of genes involved in immune response and metabolism. (d) Heat‐map of chromatin accessibility of the top 100 OCRs that discriminates cells of the monocyte, myeloid and DC lineages [cell clusters coloured as in (b)]. OCRs‐associated genes discriminating these lineages are predominately metabolic (examples indicated on heatmap). (e) Genome tracks showing IRF5/OCRs overlap occurring at the *Hk2* transcription start site (TSS) (box) in cells of the monocyte (red), myeloid (green) and DCs (blue) lineages. A IRF5/OCRs found in lung macrophages corresponding to a *Hk2* transcript variant is highlighted (*). (f,g) Accessibility of OCRs at the Hk2 TSS across immune cells (f) and in monocyte, myeloid and DCs lineages across tissue origin (g). ****P < 0·0001, by Fisher’s exact test.

To determine the role of IRF5 in controlling macrophage metabolism we first assessed IRF5 binding to DNA. To achieve this we compared IRF5 ChIP‐Seq data generated from BMDMs after 2 h of LPS exposure [9] to OCRs across immune cell types. We found that IRF5 peaks overlapped 2912 OCRs and could discriminate cells of the myeloid, monocyte and DC lineages from other immune cells based on the accessibility of these OCRs (Fig. 1a,b). Consistent with multiple published studies we found that IRF5 played a key role in immunoregulatory processes [7, 9, 13, 14]. Interestingly, we found IRF5/OCRs‐associated genes enriched for regulation of multiple metabolic processes (Fig. 1c), including lipid metabolism (e.g. *Ptgs2*), glycolysis (e.g. *Idh2*, *Hk2*) and protein metabolism (Fig. 1d). We focused on *Hk2* and found IRF5/OCRs overlaps occurred at the *Hk2* TSS (Fig. 1d) and confirmed that accessibility at *Hk2* could discriminate myeloid, monocyte and DCs lineages (Fig. 1e), with the greatest increase in accessibility occurring in macrophages and DCs originating from the gut and lung (Fig. 1f).

### IRF5 controls AM metabolic responses to Toll‐like receptor (TLR)‐3 activation

**Fig. 2:**
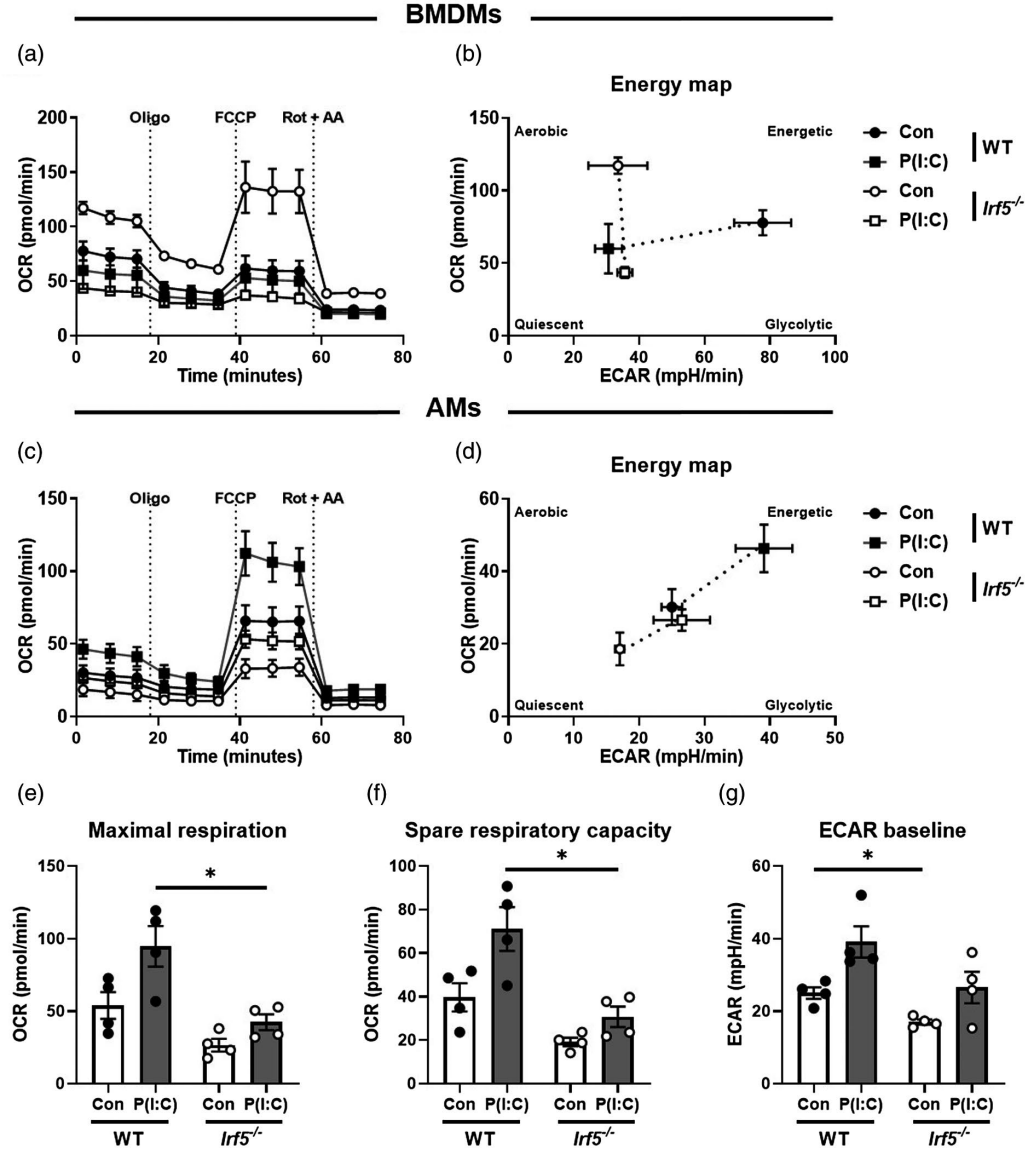
Interferon regulatory factor 5 (IRF5) controls airway macrophage (AM) metabolic responses to Toll‐like receptor (TLR)‐3 activation. (a) Analysis of the oxygen consumption rate of wild‐type (WT) or *Irf5^−/−^* macrophage–colony‐stimulating factor) (M‐CSF)‐differentiated bone marrow‐derived macrophages (BMDMs), stimulated with poly(I:C) or vehicle controls, during mitochondrial stress tests, assessed with sequential injection (dashed vertical lines) of the mitochondrial adenosine triphosphate (ATP)‐synthase inhibitor oligomycin (Oligo), the mitochondrial uncoupler carbonyl cyanide‐4‐(trifluoromethoxy)phenylhydrazone (FCCP) and inhibitors of the electron transport‐chain complexes I and III, rotenone and anti‐mycin A (Rot + AA). (b) Energy map of WT or *Irf5^−/−^* BMDMs, indicating four possible energy states; quiescent: the cell is not predisposed towards OxPhos or glycolysis; energetic: the cell utilizes both metabolic pathways; aerobic: the cell predominantly utilizes OxPhos; glycolytic: the cell predominantly utilizes glycolysis. (c) Analysis of the oxygen consumption rate of wild‐type (WT) or *Irf5^−/−^* AMs, stimulated with poly(I:C) or complete RPMI controls, during mitochondrial stress tests. (d) Energy map of WT or *Irf5^−/−^* AMs. (e) Maximal respiration of WT or *Irf5^−/−^* AMs during mitochondrial stress test, defined as the maximal oxygen consumption rate attained after addition of FCCP. (f) Spare respiratory capacity of WT or *Irf5^−/−^* AMs during mitochondrial stress test, calculated by subtraction of basal from maximal oxygen consumption rates. (g) Basal extracellular acidification rate of WT or *Irf5^−/−^* AMs. Data shown are representative of at least two individual mitochondrial stress test assays with three to four per group and are presented as mean ± standard error of the mean (s.e.m.), *P < 0·05, by Mann–Whitney *U*‐test.

Because IRF5 binds regions of open chromatin at several metabolism‐associated genes (Fig. 1) and chromatin accessibility was high in myeloid cells from gut and lung (Fig. 1f,g), we next considered whether this interaction had a functional consequence. To this end we aimed to study the cellular metabolism of WT and *Irf5^−/−^* BMDMs and AMs using a mitochondrial stress test which also provides information on key parameters of glycolysis in addition to OxPhos. We quantified both ECAR and OCR in M‐CSF (M2)‐differentiated WT or *Irf5^−/−^* BMDMs. Consistent with a role of IRF5 in controlling macrophage phenotype, *Irf5^−/−^* macrophages were more oxidative in comparison to WT controls (Fig. 2a,b) [15]. After poly(I:C) stimulation to mimic viral infection, both WT and *Irf5^−/−^* BMDMs became more metabolically quiescent (Fig. 2a,b). To determine whether IRF5 regulation had distinct consequences for AM metabolic function in comparison to BMDMs, we next investigated the metabolic profile of WT or *Irf5^−/−^* AMs *ex vivo*. After poly(I:C) stimulation *ex vivo*, WT AMs had enhanced mitochondrial oxygen consumption rates (Fig. 2c,d) compared to *Irf5^−/−^* AMs, concomitant with markedly increased maximal respiration (Fig. 2e) and spare respiratory capacity (SRC, the difference between maximal oxygen consumption, and the initial basal level, Fig. 2f), indicative of increased OxPhos. Interestingly, *Irf5^−/−^* AMs had reduced ECAR at baseline (Fig. 2g and Supporting information, Fig. S2B). Overall, unstimulated *Irf5^−/−^* AMs or those stimulated with poly(I:C) had substantially lower metabolic activity than WT controls. Thus, TLR‐3 activation in WT AMs resulted in an energetic phenotype, indicating utilization of both glycolysis and OxPhos; in contrast, *Irf5^−/−^* AMs were relatively quiescent at baseline and failed to activate metabolically (Fig. 2d).

### IRF5 controls pulmonary metabolic response to influenza infection

**Fig. 3:**
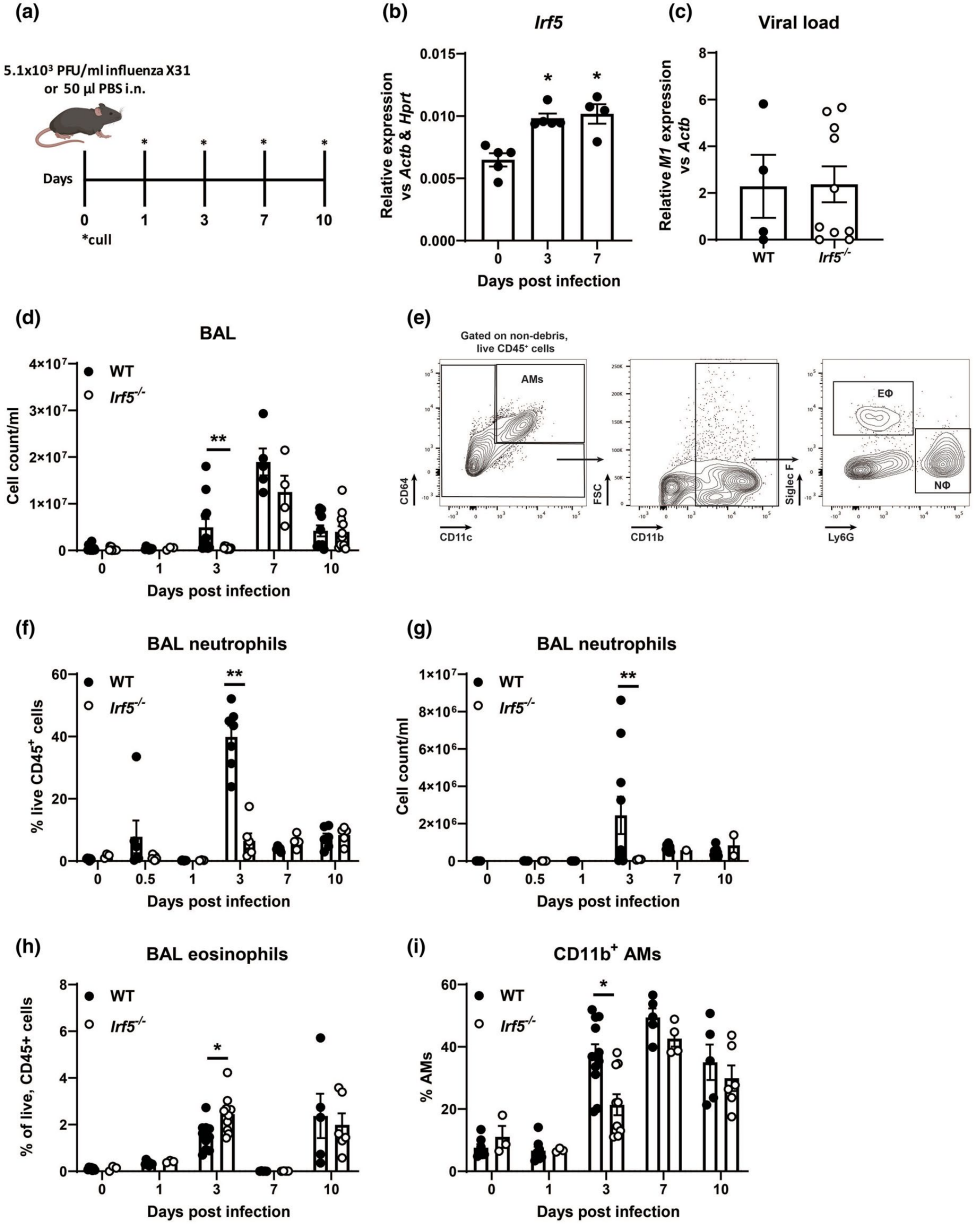
Interferon regulatory factor 5 (IRF5) controls myeloid cell recruitment in response to influenza infection. (a) Experimental design of influenza challenge model. (b) Expression of *Irf5* in whole lung homogenates. (c) Viral copy numbers were assessed in the lungs of wild‐type (WT) or *Irf5^−/−^* mice. (d) Total numbers of leucocytes recovered from the bronchoalveolar lavage (BAL) of influenza‐challenged mice or controls. (e) Gating strategy for the identification of airway macrophages (AMs), neutrophils (NΦ) and eosinophils (EΦ). Proportions (f) and numbers (g) of neutrophils recovered from the BAL of WT or *Irf5^−/−^* flu‐infected mice. Data shown are representative of at least two individual experiments with three to 14 per group and are presented as mean ± standard error of the mean (s.e.m.), *P < 0·05, **P < 0·01, by Mann–Whitney *U*‐test.

**Fig. 4:**
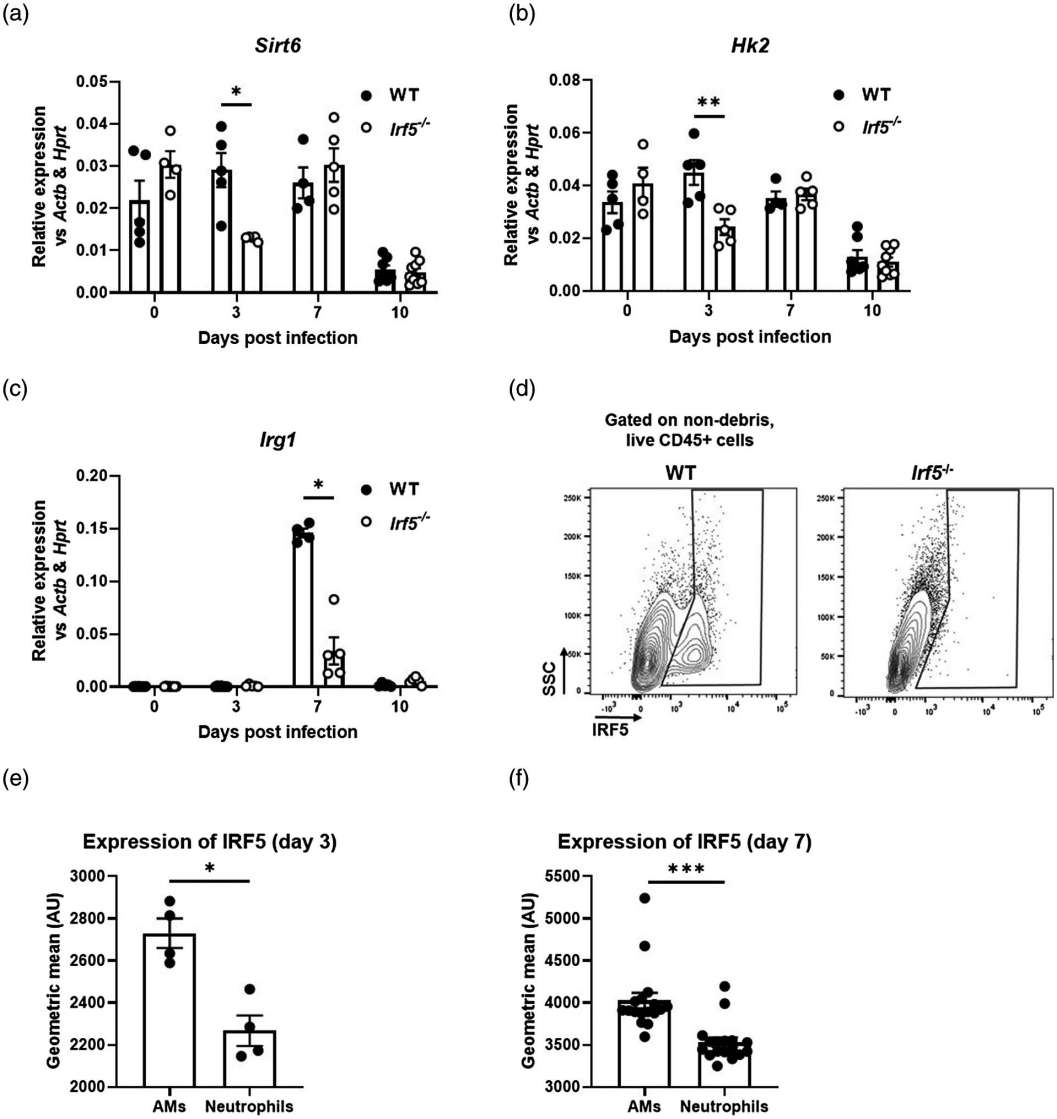
Interferon regulatory factor 5 (IRF5) controls pulmonary anti‐viral and metabolic response to influenza infection. Expression of *Sirt6* (a), *Hk2* (b) and *Irg1* (c) in the lungs of wild‐type (WT) or *Irf5^−/−^* mice. (d) Representative plots showing IRF5^+^ populations in bronchoalveolar lavage (BAL) of WT or *Irf5^−/−^* mice at day 3 post‐infection. Expression of IRF5 in BAL airway macrophages (AMs) or neutrophils at days 3 (e) or 7 (f) post‐infection. Data shown are representative of at least two individual experiments with three to 14 per group and are presented as mean ± standard error of the mean (s.e.m.), *P < 0·05, **P < 0·01, by Mann–Whitney *U*‐test.

In order to determine the functional consequences of IRF5 controlling AM metabolic phenotype upon viral infection *in vivo*, adult C57BL/6 WT and *Irf5^−/−^* mice were infected with H3N2 IAV. Intranasal infection with IAV (Fig. 3a) resulted in significantly increased *Irf5* expression in whole lung homogenates of WT mice 3 and 7 days post‐infection (p.i., Fig. 3b). Following infection, comparable viral loads were detected in WT and *Irf5*‐deficient mice (Fig. 3c). However, in the absence of IRF5, there was reduced cellular infiltrate in the BAL at day 3 p.i. (Fig. 3d); *Irf5‐*knock‐out mice had reduced neutrophilic infiltrate in terms of both proportions (Fig. 3e,f) and numbers (Fig. 3g). Although eosinophils were a relatively rare population, their numbers were increased at day 3 p.i.. Based on our observation of enriched co‐localization of IRF5 with OCRs at metabolism‐associated genes such as *Hk2* (Fig. 1), we next assessed how IRF5 controls gene expression of *Hk2* and other key metabolism enzyme genes upon viral infection *in vivo*. Interestingly, Sirtuin 6 (*Sirt6*, Fig. 4a) and *Hk2* (Fig. 4b), both involved in glucose metabolism, were down‐regulated at day 3 p.i. in *Irf5^−/−^* mice compared to controls. Furthermore, inflammatory response gene‐1 (*Irg1*; Fig. 4c) was down‐regulated in the lungs of *Irf5^−/−^* mice at day 7 p.i. *Irf5* has been shown to be highly expressed in myeloid cells, principally macrophages and neutrophils [14, 16]. Analysis of IRF5^+^ cells (Fig. 4d) in the airways of IAV‐exposed mice revealed that expression was significantly higher in AMs compared to neutrophils at both days 3 (Fig. 4e) and 7 (Fig. 4f).﻿ Examination of cellular inflammation in lung homogenates revealed that the proportions of IFN‐γ^+^CD4^+^ (Supporting information, Fig. S1A and B) and IFN‐γ^+^CD8^+^ T cells (Supporting information, Fig. S1C) peaked between days 7 and 10 p.i. in WT mice and IRF5 deficiency had no influence on the proportions of these populations. IRF5 deficiency resulted in significantly increased proportions of IL‐13^+^CD4^+^ T cells at day 10 p.i. compared to WT mice (Supporting information, Fig. S1D). We also observed no significant change in peripheral IgG1 levels and decreased levels of IgG2a in *Irf5^−/−^* mice (Supporting information, Fig. S1E and F).

## Discussion

This study provides insight into how IRF5 regulates AM phenotype by directing AM metabolism in response to TLR‐3 activation. Using published data sets we showed that proinflammatory signalling induced IRF5 binding at metabolism‐associated genes such as *Hk2* in cells of the myeloid lineage. HK catalyzes the phosphorylation of glucose, the rate‐limiting first step of glycolysis, and is a key controller of cellular metabolism. HK2 is an isoform of HK, which is constitutively active, and changes in the expression levels of *Hk2* directly impact cellular metabolism [17]. Using a mouse model of influenza A infection, we have shown that *Hk2* gene expression is controlled by IRF5 during viral infection and gene expression levels are decreased in the absences of IRF5.

Cellular metabolism is tightly linked with AM phenotype. *In vitro*, macrophages may be artificially differentiated towards proinflammatory M1 or alternatively activated M2 cells, and IRF5 has been shown to be a master regulator of the M1 phenotype [6]. A well‐described feature of the M1 phenotype is a reliance upon glycolysis, whereas M2 cells are dependent upon OxPhos [15]; we found that *Irf5^−/−^* BMDMs were more oxidative than WT cells and furthermore, consistent with increased binding of IRF5 at the *Hk2* TSS (Fig. 1), glycolysis was also substantially impacted. Taken together, these data suggest an important role for IRF5 as a regulator of glycolysis in macrophages.

Although the role of metabolic reprogramming in dictating macrophage responses to TLR‐4 activation is well described, metabolic responses to TLR‐3 engagement is less well established. Ahmed *et al*. recently reported that incubation of bone marrow‐derived M1 macrophages with poly(I:C) led to a reduction in basal respiration, ATP production and SRC, and that this occurred to a lesser extent than in LPS‐derived cells [18]. Interestingly, this effect seemed to be dose‐dependent, as lower concentrations of poly(I:C) did not alter OxPhos significantly. Consistent with the study by Ahmed *et al*., we observed a reduction in OxPhos in M‐CSF‐derived BMDMs (M2) cells at a relatively high dose of poly(I:C). While AMs moderately increased OxPhos after TLR‐3 activation in an IRF5‐dependent manner, in BMDMs this process appears to be independent of IRF5. Furthermore, we did not see a clear shift towards a glycolytic phenotype in poly(I:C)‐stimulated WT AMs, but rather a shift towards an energetic state, up‐regulating both glycolysis and TCA pathways in AMs but not in BMDMs. Although TLR‐3 activation in WT AMs resulted in an energetic phenotype, *Irf5^−/−^* AMs failed to activate metabolically.

Our work highlights distinct differences in metabolic responses to peripherally derived macrophages *versus* those found in the airways. The distinct metabolic responses of AMs are probably a consequence of the unique pulmonary environment [19]. In particular, there is significantly less glucose availability in the alveolar lumen in comparison to peripheral blood, and furthermore it has been shown that AMs do not readily engage glycolysis [20, 21]. In contrast, AMs highly express peroxisome proliferator‐activated receptor gamma (PPARγ), which promotes fatty acid oxidation and OxPhos [22]. In influenza models in mice, infiltrating monocyte‐derived AMs (Mo‐AMs) have been shown to be more glycolytic, in comparison to tissue‐resident AMs (TR‐AMs) [21]. Indeed, our recent work has shown that TR‐AMs more readily engage OxPhos, in comparison to recruited Mo‐AMs, in the context of lung fibrosis [23].

Forbester *et al*. recently showed that IRF5 is highly expressed in the myeloid lineage and promotes influenza‐induced inflammatory responses [18]. Consistent with these findings, *Irf5* is rapidly up‐regulated in response to IAV and *Irf5* expression is sustained throughout the time–course of infection. Additionally, this study reported decreased weight loss in *Irf5^−/−^* mice compared to controls; however, in our model we see a tendency towards increased weight loss in *Irf5^−/−^*. This disparity is probably explained by differences in the severity of the models utilized in each study (in our model we observed a maximum of 10% weight loss in WT mice at peak disease, compared to 20% in the work of Forbester *et al*.). IRF5 is highly expressed in AMs during IAV infection and IRF5‐deficiency results in alterations in AM metabolic phenotype as well as cellular immunity. While lung inflammation induces CD11b expression in AMs [24], *Irf5^−/−^* mice fail to up‐regulate CD11b expression in AMs in response to viral infection, suggesting a defect in AM responses. Additionally, IRF5‐deficiency leads to increased eosinophil and reduced neutrophil recruitment. These findings are consistent with our previous findings [14] and those by Oriss *et al*., showing that IRF5‐expressing macrophages promote a neutrophilic environment [25].

Together, our data highlight an important and hitherto under‐appreciated role for IRF5 as a metabolic regulator after TLR‐3 activation during respiratory viral infection. Interestingly, IRF5 is a key factor controlling AM phenotype and directly regulates HK2 a key enzyme in glycolysis. Further evaluation of the role IRF5 plays in regulating metabolic phenotype in different disease and tissue contexts may be important for the understanding of acute and chronic conditions and aid in the development novel therapeutic strategies.

## Disclosures

A. J. B. received, unrelated to the submitted work, consultancy fees from Devpro and Ionis pharmaceuticals and ﻿received consultancy fees/industry‐academic funding from Ammax pharmaceuticals, via his institution.

## Author contributions

A. J. B., I. A. U. and C. M. L. designed the study; G. J. A., J. I., P. McE., P. P. O., P. G., T. E. K. and A. J. B. carried out the work. A. J. B., C. M. L. and G. A. wrote the paper. All authors were involved in the interpretation of the results and in drafting and/or revising the manuscript, provided final approval and vouch for the content of the final manuscript.

## Supporting information


**Fig. S1**. (a) Gating strategy for the identification of T‐cells. (b) CD4^+^IFN‐γ^+^ T‐cells, (c) CD8^+^IFN‐γ^+^ T‐cells and (d) CD4^+^IL‐13^+^ T‐cells recovered from the lung of WT or *Irf5^−/−^* mice. Levels of serum (E) IgG1 (e) and (f) IgG2a in WT or *Irf5^−/−^* mice. Data shown are representative of at least two individual experiments with *n*=3‐8 per group and are presented as mean ± s.e.m., *P < 0·05, **P < 0·01, by Mann–Whitney *U* test.

**Fig. S2.** Analysis of the extracellular acidification rate of WT or *Irf5^−/−^* M‐CSF‐differentiated (a) BMDMs or (b) AMs, stimulated with Poly(I:C) or vehicle controls, during mitochondrial stress tests, assessed with sequential injection (dashed vertical lines) of the mitochondrial ATP‐synthase inhibitor oligomycin (Oligo), the mitochondrial uncoupler FCCP and inhibitors of the electron‐transport‐chain complex I and III, rotenone and antimycin A (Rot + AA). Data shown are representative of at least two individual mitochondrial stress test assays with *n* = 3‐4 per group and are presented as mean ± s.e.m.

## Data Availability

The data that support the findings of this study are available from the corresponding author upon reasonable request.

## References

[1] **Byrne** AJ , **Mathie** SA , **Gregory** LG , **Lloyd** CM . Pulmonary macrophages: key players in the innate defence of the airways. Thorax 2015; 70:1189–96. 26286722 10.1136/thoraxjnl-2015-207020

[2] Schneider C , Nobs SP , Heer AK *et al*. Alveolar macrophages are essential for protection from respiratory failure and associated morbidity following influenza virus infection. PLOS Pathog 2014; 10:e1004053. 24699679 10.1371/journal.ppat.1004053 PMC3974877

[3] Wijburg OLC , DiNatale S , Vadolas JIM *et al*. Alveolar macrophages regulate the induction of primary cytotoxic T‐lymphocyte responses during influenza virus infection. J Virol 1997; 71:9450–7. 9371606 10.1128/jvi.71.12.9450-9457.1997 PMC230250

[4] Byrne AJ , Powell JE , O’Sullivan BJ *et al*. Dynamics of human monocytes and airway macrophages during healthy aging and after transplant. J Exp Med 2020; 217:1–11.Available at: http://www.ncbi.nlm.nih.gov/pubmed/31917836. 10.1084/jem.20191236 PMC7062517 31917836

[5] Allden SJ , Ogger PP , Ghai P *et al*. The transferrin receptor CD71 delineates functionally distinct airway macrophage subsets during idiopathic pulmonary fibrosis. Am J Respir Crit Care Med 2019:rccm.201809‐1775OC. Available at: https://www.atsjournals.org/doi/10.1164/rccm.201809‐1775OC. 10.1164/rccm.201809-1775OC PMC6635794 31051082

[6] Krausgruber T , Blazek K , Smallie T *et al*. IRF5 promotes inflammatory macrophage polarization and T H1‐TH17 responses. Nat Immunol 2011; 12:231–8. 10.1038/ni.1990. 21240265

[7] Byrne AJ , Weiss M , Mathie SA *et al*. A critical role for IRF5 in regulating allergic airway inflammation. Mucosal Immunol 2017; 10:716–26.Available at: http://www.ncbi.nlm.nih.gov/pubmed/27759022. 27759022 10.1038/mi.2016.92 PMC5266556

[8] Van den Bossche J , O’Neill LA , Menon D . Macrophage immunometabolism: where are we (going)? Trends Immunol 2017; 38:395–406. 10.1016/j.it.2017.03.001. 28396078

[9] Saliba DG , Heger A , Eames HL *et al*. IRF5: RelA interaction targets inflammatory genes in macrophages. Cell Rep 2014; 8:1308–17. 10.1016/j.celrep.2014.07.034. 25159141 PMC4471814

[10] Yoshida H , Lareau CA , Ramirez RN *et al*. The cis‐Regulatory Atlas of the Mouse Immune System. Cell 2019; 176:897–912.e20. 10.1016/j.cell.2018.12.036. 30686579 PMC6785993

[11] Hammer Ø , Harper DAT , Ryan PD , Ryan DD , Ryan PD . PAST palenotological statistics. Palaentologia Electron 2011; 4:5–7.Available at: http://palaeo‐electronica.org.

[12] McLean CY , Bristor D , Hiller M *et al*. GREAT improves functional interpretation of cis‐regulatory regions. Nat Biotechnol 2010; 28:495–501.Available at: http://www.ncbi.nlm.nih.gov/pubmed/20436461%0Ahttp://www.pubmedcentral.nih.gov/articlerender.fcgi?artid=PMC4840234. 20436461 10.1038/nbt.1630 PMC4840234

[13] Weiss M , Blazek K , Byrne AJ , Perocheau DP , Udalova IA . IRF5 Is a specific marker of inflammatory macrophages *in vivo* . Mediators Inflamm 2013; 2013:1–9. 10.1155/2013/245804 PMC3885211 24453413

[14] Weiss M , Byrne AJ , Blazek K *et al*. IRF5 controls both acute and chronic inflammation. Proc Natl Acad Sci USA 2015; 112:11001–6. 10.1073/pnas.1506254112. 26283380 PMC4568217

[15] O’Neill LAJ , Kishton RJ , Rathmell J . A guide to immunometabolism for immunologists. Nat Rev Immunol 2016; 16:553–65. 27396447 10.1038/nri.2016.70 PMC5001910

[16] Courties G , Heidt T , Sebas M *et al*. *In vivo* silencing of the transcription factor IRF5 reprograms the macrophage phenotype and improves infarct healing. J Am Coll Cardiol 2014; 63:1556–66. 24361318 10.1016/j.jacc.2013.11.023 PMC3992176

[17] Roberts DJ , Miyamoto S . Hexokinase II integrates energy metabolism and cellular protection: Akting on mitochondria and TORCing to autophagy. Cell Death Differ 2015; 22:248–57. 10.1038/cdd.2014.173. 25323588 PMC4291497

[18] Ahmed D , Roy D , Jaworski A *et al*. Differential remodeling of the electron transport chain is required to support TLR3 and TLR4 signaling and cytokine production in macrophages. Sci Rep 2019; 9:18801. Available at: http://www.ncbi.nlm.nih.gov/pubmed/31827178. 31827178 10.1038/s41598-019-55295-4 PMC6906364

[19] Ogger P , Byrne A . Airway macrophage metabolic reprogramming during chronic lung disease. Mucosal Immunol 2020. 10.1038/s41385-020-00356-5. PMC7658438 33184475

[20] Svedberg FR , Brown SL , Krauss MZ *et al*. The lung environment controls alveolar macrophage metabolism and responsiveness in type 2 inflammation. Nat Immunol 2019; 20:571–80.Available at: http://www.nature.com/articles/s41590‐019‐0352‐y. 30936493 10.1038/s41590-019-0352-y PMC8381729

[21] Woods PS , Kimmig LM , Meliton AY *et al*. Tissue‐resident alveolar macrophages do not rely on glycolysis for LPS‐induced inflammation. Am J Respir Cell Mol Biol 2020; 62:243–55. 31469581 10.1165/rcmb.2019-0244OC PMC6993551

[22] Schneider C , Nobs SP , Kurrer M , Rehrauer H , Thiele C , Kopf M . Induction of the nuclear receptor PPAR‐γ 3 by the cytokine GM‐CSF is critical for the differentiation of fetal monocytes into alveolar macrophages. Nat Immunol 2014; 15:1026–37. 25263125 10.1038/ni.3005

[23] Ogger PP , Albers GJ , Hewitt RJ *et al*. Itaconate controls the severity of pulmonary fibrosis. Sci Immunol 2020; 5:1–14. 10.1126/sciimmunol.abc1884 PMC7116646 33097591

[24] Duan M , Steinfort DP , Smallwood D *et al*. CD11b immunophenotyping identifies inflammatory profiles in the mouse and human lungs. Mucosal Immunol 2016; 9:550–63. 10.1038/mi.2015.84. 26422753 PMC7101582

[25] Oriss TB , Raundhal M , Morse C *et al*. IRF5 distinguishes severe asthma in humans and drives Th1 phenotype and airway hyperreactivity in mice. JCI Insight 2017; 2:1–16.Available at: http://www.ncbi.nlm.nih.gov/pubmed/28515358%5Cnhttp://www.pubmedcentral.nih.gov/articlerender.fcgi?artid=PMC5436536%5Cnhttps://insight.jci.org/articles/view/91019. 10.1172/jci.insight.91019 PMC5436536 28515358

