# Supplementary figures and images for: IRF5 regulates airway macrophage metabolic responses
Source: Clin Exp Immunol. 2021 Jan 28;204(1):134–43. doi: 10.1111/cei.13573 (PMC7944363; doi:10.1111/cei.13573)

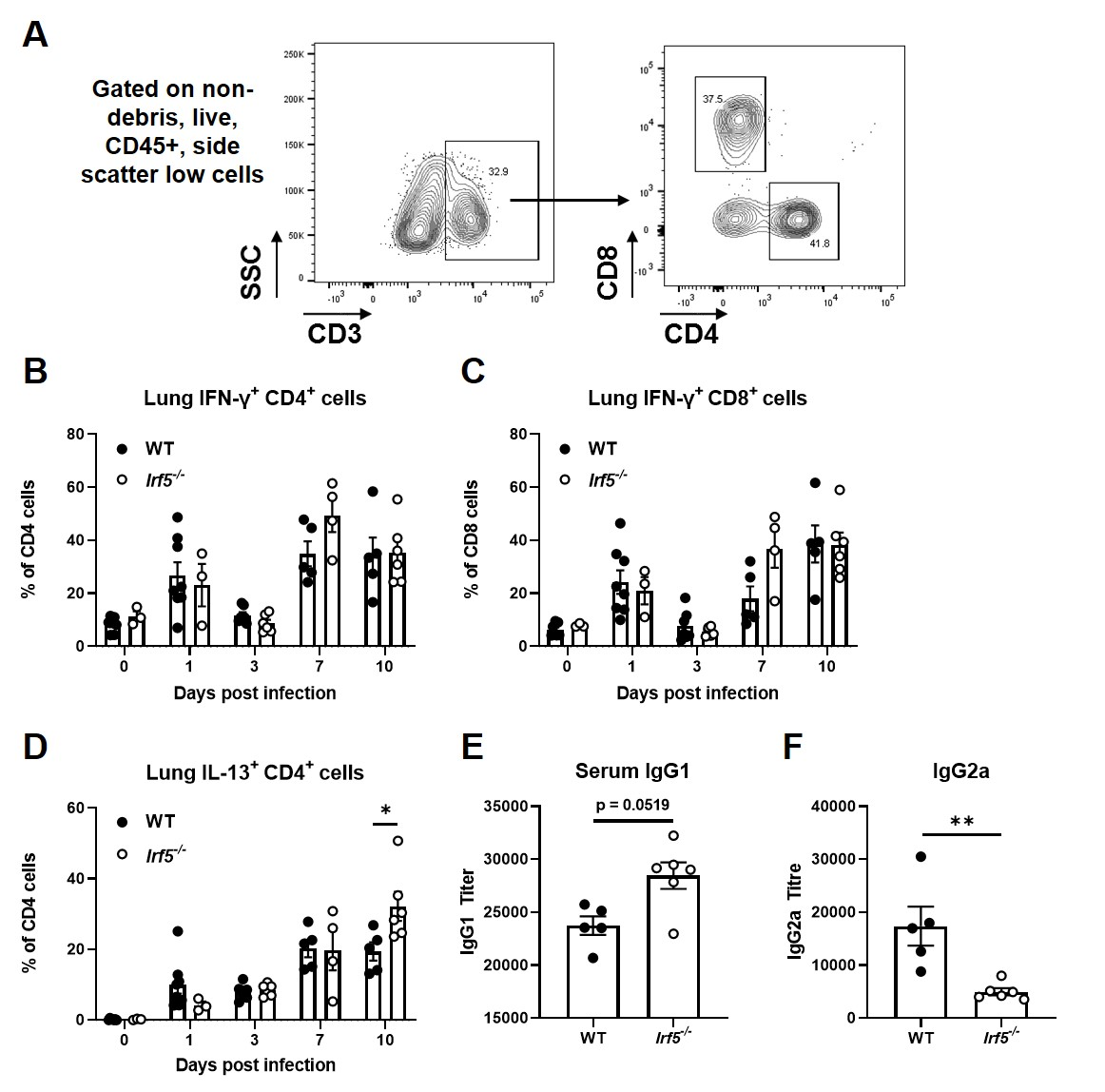

Supplement: Supplementary file 1 — Fig. S1. (a) Gating strategy for the identification of T‐cells. (b) CD4+IFN‐γ+ T‐cells, (c) CD8+IFN‐γ+ T‐cells and (d) CD4+IL‐13+ T‐cells recovered from the lung of WT or Irf5−/− mice. Levels of serum (E) IgG1 (e) and (f) IgG2a in WT or Irf5−/− mice. Data shown are representative of at least two individual experiments with n=3‐8 per group and are presented as mean ± s.e.m., *P < 0·05, **P < 0·01, by Mann–Whitney U test. [file CEI-204-134-s002.tif]

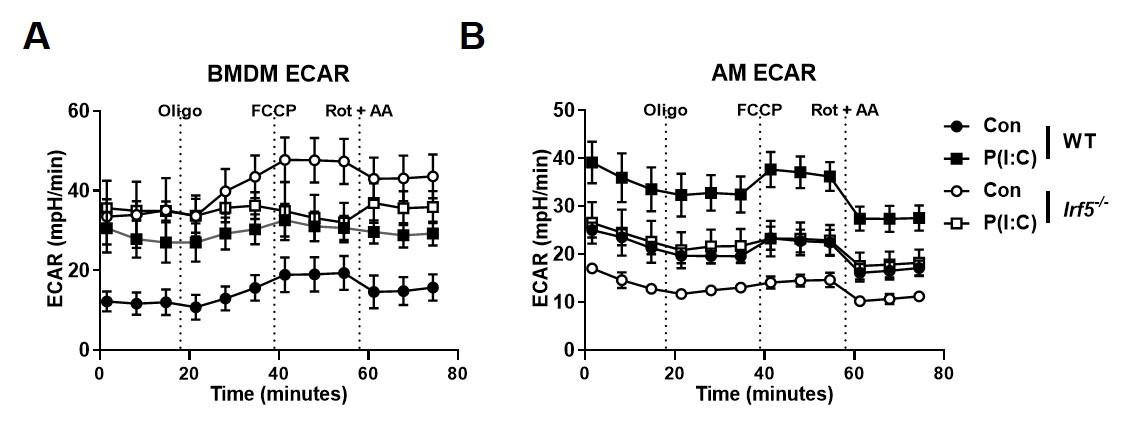

Supplement: Supplementary file 2 — Fig. S2. Analysis of the extracellular acidification rate of WT or Irf5−/− M‐CSF‐differentiated (a) BMDMs or (b) AMs, stimulated with Poly(I:C) or vehicle controls, during mitochondrial stress tests, assessed with sequential injection (dashed vertical lines) of the mitochondrial ATP‐synthase inhibitor oligomycin (Oligo), the mitochondrial uncoupler FCCP and inhibitors of the electron‐transport‐chain complex I and III, rotenone and antimycin A (Rot + AA). Data shown are representative of at least two individual mitochondrial stress test assays with n = 3‐4 per group and are presented as mean ± s.e.m. [file CEI-204-134-s001.tif]
